# Support Group for Relatives of Patients with First Psychotic Episode. When Crisis Mobilizes the System. Windows of Opportunity and Change

**DOI:** 10.1192/j.eurpsy.2025.329

**Published:** 2025-08-26

**Authors:** L. Moreno Fernández, A. R. Diez, B. R. Fernandez, J. S. Yepes, R. G. Rincon, P. C. Moreno

**Affiliations:** 1 Hospital Ramón y Cajal, Madrid, Spain

## Abstract

**Introduction:**

First psychotic episodes (FPE) have a significant impact not only on the individual who experiences them but also on their close environment, particularly their family. The onset of a FPE typically occurs in the early twenties, a stage of life where individuals are planning and developing their adult life project. The changes in family dynamics following a FPE are part of the recovery process and are considered a prognostic factor for recovery.

Since the beginning of the Comprehensive Care Program for First Psychotic Episodes, a weekly support group for families has been conducted. The group is open, lasts 90 minutes.The group has an open theme and is co-facilitated. Participants may attend after starting the psychoeducation workshop for FPE and can continue attending even after the patient has been discharged from the facility.

**Objectives:**

The aim is to analyze the predominant family figures, age range, and gender, as well as the main themes that arise and concern the families of patients with first psychotic episodes.

**Methods:**

A retrospective study with a mixed-method approach (qualitative and quantitative) is proposed, based on the narrative records collected during the weekly sessions from 2023 until June 2024.

**Results:**

The average attendance is 10.7 participants per group, mostly women (80%). The range age is from 18 to 70 years, with and average of 58 years. In terms of the roles concerning the patient: parents (95%), siblings (5%), with no representation of partners during the period analysed.

The predominant themes identified from the analysis of the collected *verbatims* include: guilt regarding the onset of the illness; the timing of recovery and managing the sense of urgency from the institution, the family, and the patient; regression in relational family dynamics; caregivers’ fear of their own death; shame and stigma; the traumatic experience of hospitalization; the distinction between behaviors associated with personality versus the illness; the rupture with identity; the fear of relapse and suicide.

Families highlight the role of the group as an emotional support, an improvement in communication strategies and bonding with the sick relative, better capacity for mentalization, and managing their own anxieties and emotions related to guilt and self-care.

**Conclusions:**

The onset of a FPE generally affects the entire family system. Past conflicts and regressive attachment dynamics are activated. Communication based on guilt harms the elements of the system. Family support groups play a crucial role in the recovery process and in helping to establish new dynamics that promote the well-being and autonomy of both the patient and caregivers. The group stands out as a source of hope, which is the main healing factor. Additionally, participants report gaining healthy coping tools and reducing their experience of isolation and stigma.

**Disclosure of Interest:**

None Declared

